# Emotion Dysregulation of Women with Premenstrual Syndrome

**DOI:** 10.1038/srep38501

**Published:** 2016-12-06

**Authors:** Mengying Wu, Ying Liang, Qingguo Wang, Yan Zhao, Renlai Zhou

**Affiliations:** 1 Department of Psychology, School of Social and Behavioral Sciences, Nanjing University, Nanjing 210023, China; 2 Beijing University of Chinese Medicine, Beijing 100029, China

## Abstract

The aim of the current study was to test whether women with premenstrual syndrome (PMS) had difficulties in emotion regulation. In Study 1, we investigated the relationship between the habitual use of emotion-regulation strategies and the severity of PMS (*n* = 230). The results showed that the severity of PMS was negatively associated with the habitual use of reappraisal, but positively associated with the habitual use of suppression. In Study 2, we first investigated the difference in the spontaneous use of suppression versus reappraisal between women with (*n* = 42) and without PMS (*n* = 42) when watching sad film clips. Then we instructed some participants (PMS group = 20, healthy group = 21) to use reappraisal to regulate their emotions induced by a second sad film clip, and the other participants were asked to watch the second film clip freely (PMS group = 22, healthy group = 21). The results showed that there was no significant difference between participants with and without PMS in the self-reported spontaneous use of emotion-regulation strategies. For participants with PMS, increases in spontaneous suppression use were associated with increases in skin conductance level (SCL), while this association was not found among participants without PMS.

The menstrual cycle is a normal physiological process that all women experience and is characterised by tightly orchestrated changes in the levels of ovarian estrogen and progesterone. Researchers have confirmed that diverse body systems (e.g., cardiovascular system^1^, central nervous system^2^, endocrine system^1^, female reproductive system^1^, and immune system^3^) are replete with estrogen receptors and that progesterone also acts on numerous tissues. Therefore, cyclically fluctuating levels of estrogen and progesterone have a significant biological effect on the female body, one with both physical and emotional ramifications^1^. Studies related to the impacts of the menstrual cycle on women’s emotional changes have been primarily conducted among patients who suffer from premenstrual syndrome (PMS) and its severe predominantly psychological form: premenstrual dysphoric disorder (PMDD)^2^. PMS is defined as a group of psychological and physical symptoms that regularly occur during the luteal phase of the menstrual cycle and resolve by the end of menstruation^3^. PMDD is characterized primarily by a cluster of mood symptoms, especially depression, tension, anxiety, irritability, and fatigue, with five or more symptoms present during the luteal phase^3^. The diagnosis of PMDD can only be made by having women prospectively monitor their symptoms for at least two consecutive symptomatic menstrual cycles^4^,^5^.

Among Chinese women, the incidence of PMDD and PMS is 2.1% and 21.1%, respectively, and the most common symptoms are irritability, breast tenderness, depression, abdominal bloating and angry outbursts^6^. Researchers found that women with PMS or PMDD had more negative mood, depression and irritability, but less positive mood as compared to healthy women during the premenstrual phase^7^, made more negative judgements in a facial discrimination task during the premenstrual phase as compared to the postmenstrual phase^8^, and had enhanced bilateral amygdala reactivity in comparison with healthy controls when exposed to emotional faces^9^. It has been indicated that most of these premenstrual complaints reported by women with PMDD are related to heightened stress-sensitivity^10^. The aetiology and pathophysiology of PMS remain unclear. Some early studies attributed the cause of PMS to abnormal and excessive secretions of the reproductive hormones^11^. However, recent studies failed to find any significant difference between women with and without PMS in the concentrations of reproductive hormones^9^,^12^, which means PMS does not seem to be due to abnormal concentrations of sex steroids. Some researchers emphasize cognitive factors in the development of PMS. It is proposed that women with PMS may be interpreting physiological changes during the premenstrual phase in a negative way, and regarding them as threatening or depriving so that they feel anxious and depressed^13^. This hypothesis was supported by the efficacy of cognitive therapy in treating PMS and PMDD^2^,^12^,^14^,^15^. Furthermore, some researchers even argue that PMS is not a fixed unitary syndrome but an ongoing process of negotiation, and reframing of symptoms as normal change can effectively reduce premenstrual distress^16^, thus implying the dysfunction of emotion-regulation based on cognitive change among women with PMS.

The process model of emotion regulation^17^ declaimed that emotion may be regulated at different points in the emotion generative process, and emotion-regulation strategies differ in when they have their primary impact on the emotion-generative process. According to this model, strategies enacted at different stages of the emotion process rely on different skills and have different consequences for emotional experience, physiology, and behavior^18^. Reappraisal and suppression are two commonly used strategies for diminishing negative emotional reactions, which have been extensively operationalized within the model^19^. Reappraisal is a type of cognitive change, and it is defined as construing a potentially emotion-eliciting situation in non-emotional terms, while suppression is a type of response modulation, and it is defined as inhibiting ongoing emotion-expressive behavior.

Researchers found that habitual emotion regulation may have an impact on individuals’ well-being and performance in emotion experience tasks^19^. The habitual use of reappraisal was related to greater positive affect, better interpersonal functioning, and higher well-being. By contrast, greater use of suppression was related to a less beneficial profile of emotional functioning^20^. Previous researches showed that cognitive reappraisal leads to the decreased expression of negative emotions and their behaviors^21^, decreased startle responses^22^ and attenuated autonomic responses^21^. Recent neuroimaging studies also revealed that the greater use of reappraisal in everyday life was related to decreased amygdala activity and increased prefrontal and parietal activity during the processing of negative emotional facial expressions^23^. Based on these findings, emotion dysregulation has long been thought to be a vulnerability factor for mood disorders^24^. However, little information is available regarding the impact of the emotion regulation on women with PMS^25^. Few studies to date evaluated the correlations between PMS and emotion regulation. With 47 papers relating to the influence of the menstrual cycle on the mental health of women, a meta-analysis conducted by Romans and his colleges indicated that 61.7% of the studies reported significant correlations between menstrual cycle and emotion regulation^26^. However, as far as we know, no study has directly tested emotion dysregulation among women with PMS.

According to previous studies, the performance of emotion regulation can be assessed in three ways^24^: (1) habitual emotion regulation; (2) spontaneous emotion regulation; (3) instructed emotion regulation. The present study adopted the paradigm of Ehring *et al*.^24^. We first assessed the relationship between habitual emotion regulation and the severity of PMS via self-report questionnaires to investigate whether the habitual use of maladaptive emotion-regulation strategy was a vulnerable factor of PMS; secondly, we used sad film clips as emotion stimuli to investigate participants’ spontaneous choice of emotion regulation strategy and their regulation efficacy in the emotion-inducing situation; thirdly, we assessed participants’ performance when instructed to use reappraisal to regulate their emotions to reveal the difference in instructed emotion regulation between women with and without PMS. Four hypotheses were tested in the current study: (1) severity of PMS is negatively associated with the habitual use of reappraisal, but positively associated with the habitual use of suppression; (2) women with PMS have higher levels of reported spontaneous emotion suppression and lower levels of reported emotion reappraisal as compared to women without PMS in the spontaneous emotion regulation task; (3) women with PMS have reduced benefit from instructed reappraisal as compared to women without PMS in the instructed emotion regulation task; (4) the difference regarding the performance of emotion regulation between women with and without PMS occurs only in the premenstrual phase.

As mentioned, menstruation is governed by estrogen and progesterone. Researchers have found that these two reproductive hormones may have an impact on the amygdala and prefrontal cortex, and thus may have effects on emotion processing and regulation^27^. In the present study, we collected each participant’s saliva sample on the day of the experiment, from which estrogen and progesterone levels were determined^28^,^29^. The hormone levels were used to confirm the phase in which each participant was on the date of the experiment, and we also investigated the relationship between emotion responses and hormone levels. Some researchers have indicated that the emotion dysregulation of individuals with mood disorders may manifest not as the failure in the regulation of self-reported negative emotion, but as the maladaptive physiological responses after emotion regulation^30^. Thus, in the current study, we used skin conductance level (SCL) as the physiological index of emotion regulation effects, which was widely used in the emotion regulation paradigm with sad film clips as emotion-inducing stimuli^31^,^32^.

## Method

### Study 1 Relationship between habitual emotion regulation and severity of PMS

#### Participants

The study was approved by the Institutional Review Board of School of the Psychology at Beijing Normal University and was in compliance with the principles of the Declaration of Helsinki. All participants signed an informed consent form prior to the start of the experiment. Two hundred and thirty healthy Chinese women were included in our sample (age: *M* = 22.0 years, *SD* = 2.2). Participants were university undergraduate or graduate students with normal, regular menstrual cycles. Their average age of menarche was 13.1 (*SD* = 1.3) years old; the average cycle length was 29.5 (*SD* = 2.8) days; the average length of their menstrual phase was 5.4 (*SD* = 1.0) days.

#### Questionnaires

##### Emotion regulation scale

Emotion regulation scale is a revised version of the Chinese version of the ERQ, which has been shown to possess good psychometric properties when assessing Chinese university students and adolescents’ habitual emotion regulation^33^. The emotion regulation scale consists of 14 items, and it assesses the habitual use of reappraisal (seven items, e.g., “I change the way I’m thinking about the situation to make myself happier”) versus suppression (seven items, e.g., “I do not let my emotion show”). Each item is rated on a scale from 1 (strongly disagree) to 7 (strongly agree). The scale has been shown to possess good psychometric properties when assessing Chinese university students and adolescents’ habitual emotion regulation^34^,^35^. Internal consistencies in the current study were satisfactory (reappraisal: α = 0.83; suppression: α = 0.80).

##### Premenstrual syndrome questionnaire

There is a lack of consensus among studies about which methods to use when collecting data about women’s premenstrual experiences^1^. One of the most common and widely used tools is the Premenstrual syndrome questionnaire^36^, Which includes 12 psychological and somatic symptoms. The severity of each symptom is rated on a four-point scale (0 = no experience of the symptom; 1 = mild experience of the symptom; 2 = the symptom has some impact on everyday work and life, but can be endured; 3 = the symptom has a serious impact on everyday work and life, and needs to be treated). Higher scores represent greater severity of symptoms. Internal consistency in the current study was satisfactory (α = 0.80).

#### Data analysis

The total scores of the reappraisal and suppression subscales in the Emotion regulation scale were calculated separately in order to assess participants’ habitual use of these two emotion-regulation strategies. The total score, positive symptom (symptoms rated above zero) number, and item score of the Premenstrual syndrome questionnaire were used as indexes of PMS severity. We calculated the correlations between PMS severity and habitual emotion regulation using SPSS 20.0 software.

#### Results

The Pearson correlation coefficients of PMS severity and habitual emotion regulation are shown in Table 1. The results showed that the habitual use of reappraisal was negatively correlated with PMS total score (*r* = −0.178, *p* = 0.007), positive symptom number (*r* = −0.158, *p* = 0.016), and the severity of emotion-related symptoms such as irritability (*r* = −0.180, *p* = 0.006), depressed mood (*r* = −0.209, *p* = 0.001), anxiety (*r* = −0.169, *p* = 0.010), and nervousness (*r* = −0.182, *p* = 0.006). These findings indicated that the greater use of reappraisal in everyday life was related to a less experience of premenstrual symptoms, especially emotion-related symptoms. The results also showed that the habitual use of suppression was not significantly correlated with PMStotal score or positive symptom number, but was positively correlated with the severity of tension (*r* = 0.172, *p* = 0.009) and insomnia (*r* = 0.174, *p* = 0.008), which revealed that the greater use of suppression in everyday life was related to a higher possibility of experiencing tension and insomnia.

### Study 2 The differences of spontaneous and instructed emotion regulation between women with and without PMS

#### Participants

Forty-two women with PMS (PMS group) and forty-two healthy women (healthy group) were screened from the sample in Study 1. The menstrual characteristics of the PMS group and healthy group are shown in Table 2. Exclusionary criteria for all participants included pregnancy, breastfeeding, current use of hormonal compounds, oral contraceptives, or psychotropic drugs, history of psychiatric disorder or alcohol and other drug abuse. All women were deemed medically and psychiatrically (except for a diagnosis of PMDD in the PMS group) healthy based on a detailed medical history and clinical interview. None of the participants were pregnant (based on urine pregnancy tests) or taking oral contraceptives, hormones or any other prescription medication. The Structured Clinical Interview for DSM-IV (SCID I, First *et al*., 1995) was conducted by a trained clinical interviewer when the women were in the follicular phase of the menstrual cycle to rule out women with a current Axis I psychiatric disorder (including substance abuse or eating disorders), except for PMDD in the PMS group^37^.

Participants in the healthy group were physically healthy women with regular menstrual cycles. They did not meet the criteria for the diagnosis of PMS, and their PMS total scores in the Premenstrual Syndrome Questionnaire^36^ and positive symptom numbers were significantly lower than those of the PMS group (*t*(82) = 4.866, *p* < 0.001, and *t*(82) = 4.843, *p* < 0.001, respectively).

The study was in compliance with the principles of the Declaration of Helsinki. All the participants signed their written informed consent to participate and the study procedures were approved by the Institutional Review Board of School of the Psychology at Beijing Normal University.

#### Date of experiment

Experimental procedures were approved by the Institutional Review Board of School of the Psychology at Beijing Normal University. All participants signed an informed consent form prior to the start of the experiment. And they would be tested twice throughout their menstrual cycles, once in the premenstrual phase (1–4 days before the onset of menstrual bleeding) and once in the postmenstrual phase (1–4 days after the offset of menstruation). We adjusted each participant’s date of experiment based on her self-reported date of the last menstrual period and her predicted date of the next menstrual period. The phase of each participant was confirmed by estrogen and progesterone levels in the saliva sample and records on the onset of the next menstrual bleeding. The phases in which the participants first came to our laboratory were randomly assigned.

#### Saliva analysis

Each participant came to our laboratory one or two days before the date of experiment to get collecting tubes and instructions for saliva collection. Participants were asked not to alter their routine sleep-wake point and not to drink alcohol on the previous night, and they were instructed to collect their saliva samples within one hour after awakening in the morning of the date of experiment. Saliva was collected without external stimulation but with muscle movement and expectoration into a 5-mL collecting tube until a 2.5-mL sample was collected. Until they finished sample collection, participants were asked to refrain from eating food, drinking fluids and brushing teeth and to rinse their mouths with water^28^.

Participants were instructed to return the samples to the laboratory immediately after collection (within 1 h); those who failed to do so were asked to store the samples in a refrigerator until they could return them to the laboratory. All the samples were frozen at −20 °C until analysis. The analyses were done in the biochemical laboratory at Beijing Protein Innovation (Beijing, China). The analyses of estrogen and progesterone were determined by competitive enzyme-linked immunosorbent assay (c-ELISA). The intra- and inter-assay coefficients of variation were below 12%.

#### Materials

##### Self-reported Emotion Inventory

Participants’ subjective emotion experiences were evaluated by the Self-reported Emotion Inventory consisting of two global terms (pleasantness and arousal) and seven emotion terms (amusement, anger, fear, disgust, sadness, surprise and peacefulness). Participants rated their feelings on nine-point Likert scales (1–9) for all the terms. Specifically, for the global terms, 1 represents extremely unpleasant and negative, 5 is neutral and 9 is extremely pleasant and positive^38^; for the emotion terms, 1 represents none and 9 represents most in my life. This inventory was also used for appropriate film stimuli selection before the experiment^38^.

##### Emotion Regulation Strategies Questionnaire

The questionnaire was used to assess the degree to which participants used reappraisal and suppression while watching the film and in the post film period^24^. It consisted of four statements: (1) I thought about the film in a way that helps me to experience less emotion; (2) I tried to adopt an unemotional attitude toward the film; (3) I tried not to let my feelings show and (4) I tried to suppress my emotions. Participants were asked to rate the statements on a scale from 1 (strongly disagree) to 9 (strongly agree). The average rating of statements (1) and (2) was used to assess the degree of reappraisal, while the average rating of statements (3) and (4) was used to assess the degree of suppression.

#### Film stimuli

Six high-definition film clips, each with a length of approximately 3 min, were used as stimuli in the current study. All the clips were from movie and TV dramas, in which the characters were all Asian. Two of the clips were used to elicit a relatively neutral emotional state and allow participants to become accustomed to our procedure, and the other four clips were used to elicit sadness. Before the experiment, 46 female graduate and undergraduate students (mean age = 21.9) were recruited to pre-test the stimuli by rating their feelings on the Self-reported Emotion Inventory after watching the film clips (Table 3). The results revealed no significant differences in overall pleasantness, arousal, or sadness for the four sad film clips, *ps* > 0.200, and the sadness scores of the four sad clips were significantly higher than those of the other six emotion terms, *ps* < 0.050. There were no significant differences in overall pleasantness, arousal, and sadness between the two neutral film clips, *ps* > 0.700, and the peacefulness scores of the two neutral clips were significantly higher than those of the other six emotion terms, *ps* < 0.050.

#### Procedure

The procedures of the two experiments conducted in the premenstrual phase and postmenstrual phase were identical. Upon arrival at the laboratory, participants were informed that the study was about emotion, and then they reviewed and signed a consent form. We then attached physiological sensors to them while they were seated in a comfortable chair in front of a computer. After that, participants were asked to find a comfortable sitting position and reminded to avoid any unnecessary movements and speech during the procedure.

First, participants were asked to relax for 3 min, after which they filled in the Self-reported Emotion Inventory. Next, they received the following instruction: “We will now be showing you a short film clip. It is important to us that you watch the film clip carefully until the end”. After that, they watched a neutral film clip. After the film clip, they filled in the Self-reported Emotion Inventory, and then they were asked to relax for another 3 min. Next, they filled in the Self-reported Emotion Inventory, and received the same instruction as before, and then, they watched the first sad film clip, which was used to assess their spontaneous emotion regulation. After the film clip, they filled in the Self-reported Emotion Inventory and the Emotion Regulation Strategies Questionnaire, and then they were asked to relax for 3 min. After that, they were asked to fill in the Self-reported Emotion Inventory and the Emotion Regulation Strategies Questionnaire again. Then, participants were divided into the reappraisal group (*n* = 41) and the watch group (*n* = 43) randomly. 20 women in the reappraisal group were from the PMS group, and the other 21 women were from the healthy group; 22 women in the watch group were from the PMS group, and the other 21 were from the healthy group. The instructions for the reappraisal group and the watch group were adapted from Ehring *et al*.^24^. Participants in the reappraisal group received the following instructions:

“We will now be showing you another short film clip. It is important to us that you watch the film clip carefully until the end. This time, please try to adopt a neutral and unemotional attitude as you watch the film. In other words, as you watch the film, try to concentrate on what you are seeing objectively. Imagine that you are a director and pay attention to the technical aspects of the film, the way in which certain moods are produced, and the cuts and camera angles used. Watch the film clip carefully, but please remember to think about what you are seeing in such a way that you don’t feel anything at all.”

Participants in the watch group received the following instructions:

“We will now be showing you a short film clip. It is important to us that you watch the film clip carefully until in the end.”

After making sure that all participants understood the meaning of the instructions, the second sad film clip began. After that, participants filled in the Self-reported Emotion Inventory and the Emotion Regulation Strategies Questionnaire. After the 3-min relaxation period, participants were asked to fill in the self-reported emotion inventory and the emotion regulation strategies questionnaire for the last time.

#### Physiological measures

Skin conductance level (SCL) was recorded at a rate of 32 samples/s with a NeXus-10-SC/GSR sensor (two finger sensor) connected to the NeXus-10 recording system with a 24-bit resolution which is able to register changes of less than 0.0001 microsiemens. We attached one sensor to the middle finger and the other sensor to the ring finger of the left hand, which was passive during the experiment. The participants were asked to avoid any movements of this hand to reduce any artifacts in the SCL from motion. Afterward, Biotrace+ software (Mind Media B.V., Netherlands) supplied with the NeXus-10 was applied to data reduction, artifact control, and computation of average SCL scores for each participant for each 3-min relaxation period and film clip.

#### Results

##### Differences between PMS group and healthy group in hormone levels across the menstrual cycle

One participant in the PMS group and one participant in the healthy group failed to collect enough saliva for analysis, so we ultimately obtained 82 participants’ (PMS group = 41; healthy group = 41) hormone levels data. The results showed that the PMS group’s mean estrogen and progesterone levels in the premenstrual phase were 4.32 pg/mL (range from 0.69 to 9.80, SD = 2.08), and 264.34 pg/mL (range from 37.75 to 804.93, SD = 122.24), respectively, while in the postmenstrual phase, mean estrogen was 3.55 pg/mL (range from 0.16 to 8.45, SD = 1.63), and mean progesterone was 89.68 pg/mL (range from 15.51 to 444.18, SD = 83.17); healthy group’s mean estrogen and progesterone in the premenstrual phase was 4.70 pg/mL (range from 2.14 to 11.99, SD = 2.22), and 241.72 pg/mL (range from 32.77 to 655.44, SD = 116.30), respectively, while in the postmenstrual phase, mean estrogen was 3.89 pg/mL (range from 1.16 to 10.36, SD = 1.97), and mean progesterone was 81.71 pg/mL (range from14.26 to 141.03, SD = 35.48).

Because the distributions of the hormone concentrations were skewed, estrogen and progesterone levels were log-transformed for analysis. A 2 (group: PMS group, healthy group) × 2 (phase: premenstrual, postmenstrual) mixed model analysis of variance (ANOVA)with the transformed data as the dependent variable was conducted. The results showed significant main effects of phase: estrogen level in the premenstrual phase was significantly higher than that in the postmenstrual phase, *F*(1, 80) = 12.341, *p* = 0.001, *η*_*p*_^*2*^ = 0.134, and as were progesterone levels, *F*(1, 80) = 62.500, *p* < 0.001, *η*_*p*_^*2*^ = 0.439. The main effects of group (*ps* > 0.300) and the interactions of group and phase (*ps* > 0.200) were not significant. These results were consistent with previous findings about patterns of salivary estrogen and progesterone across the menstrual cycle^28^,^29^,^39^, which confirmed the phase of participants.

##### Responses to neutral films of PMS group and healthy group across the menstrual cycle

A 2 (time: prefilm, postfilm) × 2 (group: PMS group, healthy group) × 2 (phase: premenstrual, postmenstrual) mixed model analysis of variance (ANOVA)with overall pleasantness scores as the dependent variable was conducted. The results showed a significant main effect of time, *F* (1, 81) =  43.746, *p* < 0.001, *η*_*p*_^*2*^ = 0.351, and the overall pleasantness scores decreased significantly after watching neutral film clips. The main effects of group (*F* (1, 81) = 0.346, *p* = 0.558, *η*_*p*_^*2*^ = 0.004) and phase (*F*(1, 81) = 0.079, *p* = 0.779, *η*_*p*_^*2*^ = 0.015) were not significant and no significant interactions were found, *ps* > 0.100.

Similarly, a 2 (time: prefilm, film) × 2 (group: PMS group, healthy group) × 2 (phase: premenstrual, postmenstrual) mixed model analysis of variance (ANOVA)with SCL as the dependent variable was conducted. Prefilm SCL referred to the mean amplitude of SCL during 3-min relax before the flim, and film SCL referred to the mean amplitude of SCL during the film. The results showed a significant main effect of time, *F* (1, 82) = 168.044, *p* < 0.001, *η*_*p*_^*2*^ = 0.672, SCL increased significantly during the film; the main effects of group (*F*(1, 82) = 0.083, *p* = 0.774, *η*_*p*_^*2*^ = 0.001) and phase (*F*(1, 82) = 0.051, *p* = 0.882, *η*_*p*_^*2*^ = 0.001) were not significant and no significant interactions were found, *ps* > 0.050.

These findings indicated that the responses to neutral films did not differ between women with and without PMS, and that the menstrual cycle did not influence their responses.

##### Differences in spontaneous emotion regulation between PMS group and healthy group across menstrual cycle

A 2 (group: PMS group, healthy group) × 2 (phase: premenstrual, postmenstrual) mixed-model analysis of variance (ANOVA)with self-reported reappraisal level as the dependent variable was conducted to investigate whether women with PMS differed from women without PMS in spontaneous reappraisal across the menstrual cycle. The results showed that the main effects of group (*F*(1, 82) = 0.011, *p* = 0.916, *η*_*p*_^*2*^ < 0.001) and phase (*F*(1, 82) = 0.116, *p* = 0.734, *η*_*p*_^*2*^ = 0.001) were not significant, neither was the interaction of the two factors, *F*(1, 82) = 0.349, *p* = 0.556, *η*_*p*_^*2*^ = 0.004.

We conducted the mixed-model ANOVA with self-reported suppression level as the dependent variable, and we also found that the main effects of group (*F*(1, 82) = 0.398, *p* = 0.530, *η*_*p*_^*2*^ = 0.005) and phase (*F*(1, 82) = 0.002, *p* = 0.965, *η*_*p*_^*2*^ < 0.001) were not significant, and that neither was the interaction of the two factors, *F*(1, 82) = 0.049, *p* = 0.825, *η*_*p*_^*2*^ = 0.001.

These findings indicated that there were no significant differences in self-reported spontaneous emotion regulation between women with and without PMS, and the menstrual cycle did not influence their self-reported spontaneous emotion regulation levels.

##### Effects of spontaneous emotion regulation and group on emotional responses (the first sad film)

We adopted the regression method used by Ehring *et al*.^24^ to test the effects of spontaneous emotion regulation and group on emotion responses to the first sad film clip.

#### Self-reported sadness score

First, we computed two residualized sadness scores in the premenstrual phase by regressing (a) the sadness scores during the film and (b) the pleasantness scores after the 3-min postfilm recovery interval on the prefilm sadness scores. Then, four different regression analyses were conducted: (1) dependent variable (DV): residualized sadness scores during the film; independent variables (IVs): group (scored as a dummy variable), self-reported reappraisal during the film (as continuous variable), and the interaction term(Group & Self-reported reappraisal); (2) DV: residualized sadness scores during the film; IVs: group, self-reported suppression, interaction term (group & self-reported suppression); (3) DV: residualized sadness scores after the 3-min postfilm recovery interval; IVs: group, self-reported reappraisal, interaction term (group & self-reported reappraisal); (4) DV: residualized sadness scores after the 3-min postfilm recovery interval; IVs: group, self-reported suppression, interaction term (group & self-reported suppression). The self-reported sadness scores in the postmenstrual phase were analyzed in the same way.

In all analyses, the main effects of group and the interaction terms were nonsignificant, *ps* > 0.090. The results showed that self-reported reappraisal levels had no significant effects on the residualized sadness scores, *ps* > 0.300. The analyses revealed that during the postmenstrual phase, high levels of suppression during the film were related to high sadness scores during the film, *t*(80) = 2.703, *p* = 0.008, *β* = 0.465, and high levels of suppression during the recovery interval were related to high levels of sadness scores after recovery, *t*(80) = 3.210, *p* = 0.002, *β* = 0.460. However, the effects of suppression levels on sadness scores were nonsignificant during the premenstrual phase, *ps* > 0.090.

#### SCL

The regression method^24^ was also used to analyse the effects of spontaneous emotion regulation and group on SCL during the first sad film clip. In all analyses, the main effects of group and spontaneous emotion regulation levels were nonsignificant, *ps* > 0.060. When looking at residualized SCLs during the film in the premenstrual phase, the interaction between group and self-reported suppression was significant, *t* (80) = 2.050, *p* = 0.044, *β* = 0.196. Follow-up tests showed that high levels of suppression were related to high SCL for participants with PMS, *t*(40) = 1.990, *p* = 0.053, *β* = 0.143, but not for those without PMS, *t*(40) = −0.835, *p* = 0.409, *β* = −0.053.

##### Effects of hormone levels and group on emotional responses

The same regression method was adopted. The residualized sadness scores calculated above were used as dependent variables, and PMS group, estrogen and progesterone levels (log-transformed), and the interaction terms (group & hormone levels) were used as independent variables. In all analyses, no significant effects were found, *ps* > 0.100. When looking at residualized SCLs, the effects were also nonsignificant in all analyses, *ps* > 0.050.

Effects of group, phase and instructed emotion regulation on emotional responses (the second sad film).

#### Manipulation check

A 2 (condition: watch, reappraisal) × 2 (group: PMS group, healthy group) × 2 (phase: premenstrual, postmenstrual) mixed-model analysis of variance (ANOVA)with self-reported reappraisal level during the second sad film as the dependent variable was conducted. The results showed that participants in the reappraisal group reported higher levels of reappraisal as compared to participants in the watch group, *F* (1, 80) = 76.032, *p* < 0.001, *η*_*p*_^*2*^ = 0.487. The main effect of phase was nonsignificant, *F* (1, 80) = 0.012, *p* = 0.915, *η*_*p*_^*2*^ < 0.001, and none of the interactions were significant, *ps* > 0.08. Taken together, the results showed the experimental induction of reappraisal during the second sad film was successful. Participants with and without PMS did not differ in self-reported reappraisal level when they were instructed to use this strategy, and the menstrual cycle didn’t influence participants’ self-reported efforts in using reappraisal when they were instructed to do so.

#### Self-reported sadness score

A 2 (condition: watch, reappraisal) × 2 (group: PMS group, healthy group) × 2 (phase: premenstrual, postmenstrual) × 3 (time: prefilm, film, postfilm) mixed-model analysis of variance (ANOVA)with self-reported sadness score during the second sad film as the dependent variable was conducted. The results showed that the main effect of time was significant, *F*(2, 160) = 103.808, *p* < 0.001, *η*_*p*_^*2*^ = 0.565; self-reported sadness score during the film was significantly higher than that during the prefilm baseline and postfilm recovery interval, *ps* < 0.001, while the sadness score during prefilm baseline and postfilm recovery interval didnot differ significantly, *p* = 0.143. The results also revealed a significant main effect of condition, *F*(1, 80) = 13.512, *p* < 0.001, *η*_*p*_^*2*^ = 0.144, and a significant interaction between time and condition, *F*(2, 160) = 13.707, *p* < 0.001, *η*_*p*_^*2*^ = 0.146. Follow-up tests showed that whereas participants in the two conditions didn’t significantly differ at the prefilm baseline and postfilm recovery interval, *ps* > 0.050, participants in the reappraisal condition reported significantly less sadness during the film than those in the watch condition, *p* < 0.001. All other main effects and interactions were nonsignificant, *ps* > 0.050.

#### SCL

**Figure 1:**
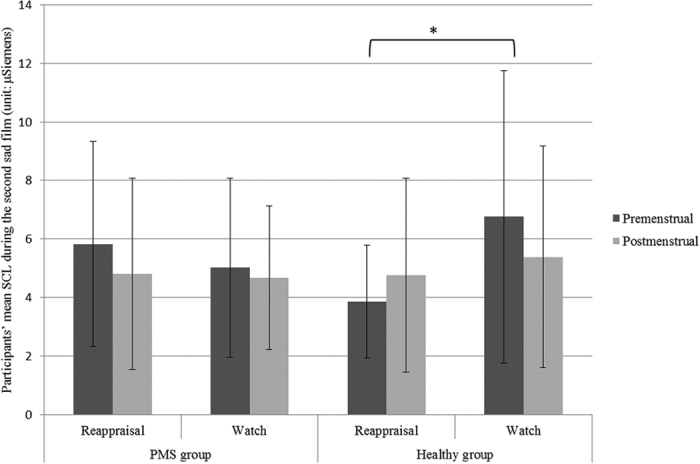
Effects of group, phase and instructed reappraisal on SCL during the second sad film.

Participants’ SCL data during the second sad film are shown in Table 4. A 2 (condition: watch, reappraisal) × 2 (group: PMS group, healthy group) × 2 (phase: premenstrual, postmenstrual) × 2(task: sad vs rest state) mixed-model analysis of variance (ANOVA) was performed on the data. The results showed that the main effects of condition (F(1, 80) = 1.436, p = 0.234, η_p_^2^ = 0.018) and group (F(1, 80) = 0.044, p = 0.835, η_p_^2^ = 0.001) were not significant, and neither was the main effect of phase, F(1, 80) = 0.598, p = 0.442. The interaction between group and condition was significant, F(1, 80) = 6.687, p = 0.012, η_p_^2^ = 0.077. Follow-up tests revealed that participants without PMS in the reappraisal condition demonstrated lower SCL than those in watch condition, *p* = 0.017, whereas participants with PMS in reappraisal condition and watch condition didnotdiffer in SCL, *p* = 0.518 (see Fig. 1). All other interactions were nonsignificant, *ps* > 0.050.

## Discussion

This study aimed to test the idea that PMS vulnerability is linked to alterations in emotion regulation. Four hypotheses regarding emotion dysregulation among women with PMS were derived, and they were each partly supported by our findings.

### Habitual emotion regulation and PMS

We hypothesised that the severity of PMS was negatively associated with habitual use of reappraisal, but positively associated with the habitual use of suppression. Our findings of the survey conducted in Study 1 supported this idea.

Reappraisal and suppression are two important forms of emotion regulation. Their differing consequences have long been investigated by experimental^18^ and individual-difference studies^40^. These studies find reappraisal is often more effective than suppression. Reappraisal decreases emotion experience and physiological arousal. By contrast, suppression fails to decrease emotion experience, and may even increase physiological responding for suppressors (for a review, see Gross, 2002). Gross and John tested the relationship between the habitual use of emotion-regulation strategies and affect, well-being, and social functioning^20^. They found that using reappraisal was associated with greater positive emotion, better well-being, and better interpersonal functioning, whereas using suppression was associated with greater negative emotion, worse welling-being, and worse interpersonal functioning. These findings support the idea that reappraisal is an adaptive emotion-regulation strategy, and the habitual use of reappraisal is beneficial to well-being, whereas suppression is a maladaptive strategy, and the habitual use of suppression impairs well-being. Our findings in Study 1 provide further evidence for this idea. Habitual emotion regulation may influence women’s premenstrual affect and physiologcial responses. This result suggests that an intervention of habitual emotion regulation may help to relieve PMS.

### Spontaneous emotion regulation and PMS

As for hypothesis 2, no significant group differences have been found between participants with and without PMS for self-reported spontaneous emotion regulation in Study 2. An overview of previous studies make it obvious that the association between mood disorders and emotion dysregulation is still unclear. Gruber, Harvey and Gross examined the use of specific emotion-regulation strategies among individuals with bipolar disorder (BD) and healthy controls^41^. The results indicated that the individuals with BD made greater use of spontaneous suppression and reappraisal compared to the healthy controls. Ehring *et al*. tested the association between depression vulnerability and difficulties with spontaneous emotion regulation^24^. They found that the recovered-depressed participants reported having spontaneously used suppression during sadness-inducing film more often than healthy controls, whereas the two groups ‘didnot differ regarding spontaneous use of reappraisal. Some researchers argue that there may be specificity in the relationship between certain emotion-regulation strategies and different psychopathologies. According to the findings of a meta-analysis study, suppression was more strongly related to anxiety^42^. However, no other studies to date have directly tested spontaneous emotion regulation among women with PMS, and our study only tested the spontaneous use of reappraisal and suppression. Thus, future research is needed to determine whether women with and without PMS differ in spontaneous use of other emotion-regulation strategies.

It should be noted that high levels of spontaneous suppression were found to be related to high SCL during the first sad film for participants with PMS but not for those without PMS in the regression analysis. Previous studies have indicated that suppression increases physiological response including SCL for suppressors (for a review, see Gross, 2002), whereas adaptive emotion-regulation strategies like reappraisal and acceptance help decrease physiological responding induced by negative emotion^8^,^30^,^32^. Although we did not find group differences in self-reported spontaneous emotion regulation, the results of the regression analysis may indicate that spontaneous suppression has a greater impact on women with PMS than those without PMS.

### Instructed emotion regulation and PMS

Our third hypothesis predicted that women with PMS have reduced benefit from instructed reappraisal as compared to women without PMS. The hypothesis was supported as the effects of instructed reappraisal differed between the groups. Whereas participants without PMS in the reappraisal condition demonstrated lower SCL than those in the watch condition, instructed reappraisal couldn’t help participants with PMS decrease SCL evoked by the second sad film. Similarly, Aldao and Mennin examined the implementation of reappraisal by participants with and without a diagnosis of generalized anxiety disorder (GAD) while watching emotion-eliciting film clips^42^. They found that although self-reported reappraisal levels did not differ between groups, implementation of reappraisal produced differential effects in the physiological (but not subjective) domain across diagnostic groups. Participants with GAD seemed not to benefit from the use of reappraisal as the healthy controls did. Researchers suggested that these findings help to delineate the specific emotion regulation profile associated with GAD^42^. In our current work, the implementation of an adaptive emotion-regulation strategy (i.e. reappraisal) seemed to help women with PMS decrease subjective negative emotions, but have nothing to do with their physiological arousal, which can be seen as the specific emotion profile associated with PMS.

### Menstrual cycle, emotion regulation, and PMS

PMS is a group of symptoms that regularly occur during the luteal (premenstrual) phase and resolve by the end of menstruation^2^, so we hypothesised that the differences regarding the performance of emotion regulation between women with and without PMS manifested only in the premenstrual phase. However, we did not find any significant main effects or interactions related to phase in Study 2. Some researchers suggest that a trait-like negative bias in the perception of life events may play an important role in the emergence of PMS, and that negative bias exists throughout the whole menstrual cycle, not only during the premenstrual phase^13^. Some therapists working with women reporting PMS also argue that PMS evolve in the context of an ongoing interaction between internal experiences, perceptions, reactions, relationships, and cultural expectations across the menstrual cycle^16^. Our finding that the emotion dysregulation of women with PMS existed throughout the entire menstrual cycle also supports this idea. We speculate that women with PMS may have more difficulties in coping with the menstrual changes due to their trait-like emotion dysregulation, which may in turn aggravate their symptoms.

### Hormones, emotion regulation, and PMS

This study examined the differences between women with and without PMS in the levels of estrogen and progesterone and the influence of hormonal levels on emotional responding. No significant results were found. Although some researchers believe that sex steroids get involved in the triggering of PMS, there is little support for the long-held view that PMS is due to a hormone deficiency, and differences between women with and without PMS in hormonal levels across the menstrual cycle are seldom found^12^,^43^. Overall, studies that have investigated women during different phases of the menstrual cycle suggest that progesterone increases amygdala reactivity, whereas estrogen may have the opposite effect on amygdala reactivity. In addition, the influence of hormones appears to be dose-dependent^44^. Progesterone concentration similar to that observed during the luteal (i.e. premenstrual) phase led to an increase in amygdala reactivity to threatening faces^45^, whereas a higher progesterone concentration, similar to that observed during pregnancy, led to a reduction in amygdala activity during the memorization of happy and neutral faces^42^. The nonlinear correlation between hormonal levels and emotional responding may be the reason why we didn’t find the influence of hormone on emotional responding using linear regression analysis. Thus more advanced analysis methods are required to clarify the interactions between sex hormones and emotional responding in the future studies.

## Conclusions

According to our results, women with PMS appear to have a trait-like emotion dysregulation throughout the menstrual cycle. Greater use of reappraisal in everyday life is related to less experience of premenstrual symptoms, especially those emotion-related symptoms, whereas greater use of suppression in everyday life is related to higher possibility of experiencing premenstrual symptoms. Spontaneous suppression of negative emotions increases physiological arousal of women with PMS, and instructed reappraisal of negative emotions can’t help women with PMS decrease physiological arousal. These results help delineate the specific emotion regulation profile associated with PMS.

## Additional Information

**How to cite this article**: Wu, M. *et al*. Emotion Dysregulation of Women with Premenstrual Syndrome. *Sci. Rep.* **6**, 38501; doi: 10.1038/srep38501 (2016).

**Publisher's note:** Springer Nature remains neutral with regard to jurisdictional claims in published maps and institutional affiliations.

## Figures and Tables

**Table 1:** Correlations of PMS severity and habitual use of suppression and reappraisal (*n* = 230).

	Suppression	Reappraisal
PMS total score	0.081	−0.178**
Positive symptom number	0.104	−0.158*
Irritability	−0.007	−0.180**
Depressed mood	0.093	−0.209**
Anxiety	0.73	−0.169*
Abdominal distension	−0.75	0.031
Inattention	0.066	−0.123
Drowsiness	−0.015	−0.053
Tension	0.172**	−0.055
Fidget	0.082	−0.063
Migraine	−0.038	−0.062
Insomnia	0.174**	−0.042
Swelling of hands and feet	−0.042	−0.076
Nervousness	0.044	−0.182**

Note: *p < 0.05, **p < 0.01.

**Table 2:** Demographic and menstrual characteristics of the sample in study2 (*M* ± *SD*).

	PMS group (*n* = 42)	Healthy group (*n* = 42)	*t*
PMS total score	8.26 ± 3.43	4.98 ± 2.72	4.866*
PMS positive symptom number	6.79 ± 2.30	4.48 ± 2.08	4.843*
Age	21.86 ± 2.27	22.31 ± 2.54	−0.860
Age of menarche	12.75 ± 1.19	12.86 ± 1.35	−0.386
Length of menstrual cycle	29.25 ± 2.44	29.45 ± 2.71	−0.360
Length of menstrual flow	5.40 ± 0.89	5.08 ± 0.96	1.593

Note: *p < 0.001.

**Table 3:** Mean (standard deviation) of the scores for film stimuli on all dimensions in the self-reported emotion inventory.

Film clip	Pleasantness	Arousal	Amusement	Anger	Fear	Sadness	Peacefulness	Disgust	Surprise
Neutral clip 1 (*n* = 25)	3.00 (1.73)	2.76 (2.37)	2.76 (1.79)	1.32 (0.90)	1.20 (0.50)	1.28 (0.74)	5.84 (2.56)	2.64 (2.20)	2.08(2.00)
Neutral clip 2 (*n* = 25)	2.96 (1.81)	2.64 (1.75)	2.76 (1.74)	1.68 (1.44)	1.80 (1.76)	2.12 (2.05)	5.96 (2.72)	2.32 (2.08)	2.08 (1.47)
Sad clip 1 (*n* = 21)	1.81 (1.33)	5.86 (1.90)	1.76 (1.26)	2.05 (1.63)	2.57 (1.96)	6.76 (1.92)	3.29 (1.85)	1.48 (0.93)	2.24 (1.81)
Sad clip 2 (*n* = 21)	1.81 (0.93)	5.67 (1.88)	2.00 (1.14)	1.67 (1.11)	2.67 (2.18)	6.19 (2.09)	4.19 (2.29)	1.62 (1.20)	1.86 (1.28)
Sad clip 3 (*n* = 21)	2.10 (1.58)	6.38 (1.94)	1.90 (1.26)	1.52 (1.21)	2.19 (1.83)	7.14 (1.59)	3.43 (1.72)	1.71 (1.19)	2.38 (1.77)
Sad clip 4 (*n* = 21)	1.76 (1.37)	6.10 (2.19)	1.71 (0.85)	2.24 (1.87)	2.57 (1.60)	6.67 (2.31)	3.71 (2.03)	2.19 (1.75)	1.86 (1.24)

**Table 4:** Participants’ SCLs during the second sad film across the menstrual cycle (*M* ± *SD*).

Phase	PMS group		Healthy group	
	Reappraisal (*n* = 20)	Watch (*n* = 22)	Reappraisal (*n* = 21)	Watch (*n* = 21)
Premenstrual	5.83 ± 3.49	5.02 ± 3.06	3.86 ± 1.93	6.76 ± 4.99
Postmenstrual	4.81 ± 3.26	4.68 ± 2.46	4.76 ± 3.31	5.39 ± 3.78

Note: The unit of SCL is μSiemens.
